# Genome-Wide Association Study Provides Insights into Important Genes for Reproductive Traits in Nelore Cattle

**DOI:** 10.3390/ani11051386

**Published:** 2021-05-13

**Authors:** Ana Paula Sbardella, Rafael Nakamura Watanabe, Rebeka Magalhães da Costa, Priscila Arrigucci Bernardes, Larissa Graciano Braga, Fernando Sebastián Baldi Rey, Raysildo Barbosa Lôbo, Danísio Prado Munari

**Affiliations:** 1 Departamento de Engenharia e Ciências Exatas, Universidade Estadual Paulista, Jaboticabal 14884-900, São Paulo, Brazil; paulasbardella@gmail.com (A.P.S.); rafael.nakamura@unesp.br (R.N.W.); rebeka.magalhaes@gmail.com (R.M.d.C.); larissa.graciano@unesp.br (L.G.B.); 2 CRIARIS Pecuária Inteligente, Goiânia 74780-712, Goiás, Brazil; 3 Departamento de Zootecnia e Desenvolvimento Rural, Universidade Federal de Santa Catarina, Florianópolis 88034-000, Santa Catarina, Brazil; priscila.arrigucci@ufsc.br; 4 Departamento de Zootecnia, Universidade Estadual Paulista, Jaboticabal 14884-900, São Paulo, Brazil; fernandobaldiuy@gmail.com; 5 Departamento de Genética, Universidade de São Paulo, Ribeirão Preto 14040-030, São Paulo, Brazil; raysildo@ancp.org.br; 6 Associação Nacional de Criadores e Pesquisadores (ANCP), Ribeirão Preto 14020-230, São Paulo, Brazil

**Keywords:** beef cattle, WssGWAS, reproduction, candidate genes

## Abstract

**Simple Summary:**

In this study, we investigated the association between single nucleotide polymorphisms (SNPs) and reproductive traits in order to identify candidate genes and biological pathways associated with these traits in Nelore beef cattle. The genome-wide association analysis revealed genomic regions that could explain part of the genetic variance of the studied traits. The results revealed genes with important functions for reproductive traits, such as fertility and precocity. Some genes were associated with more than one trait, being important for reproductive efficiency. The identification of candidate genes that were associated with the studied traits as well as genes enriched in the functional terms and pathways may be useful for exploring the genetic architecture underlying reproductive traits and may be used in Nelore breeding programs.

**Abstract:**

The identification of genomic regions associated with reproductive traits as well as their biological processes allows a better understanding of the phenotypic variability of these traits. This information could be applied to animal breeding programs to accelerate genetic gain. The aim of this study was to evaluate the association between single nucleotide polymorphisms (SNP) with a scrotal circumference at 365 days of age (SC365) and at 450 days of age (SC450), gestation length (GL) as a calf trait, age at first calving (AFC), accumulated productivity (ACP), heifer early calving until 30 months (HC30), and stayability (STAY) traits, in order to identify candidate genes and biological pathways associated with reproductive traits in Nelore cattle. The data set consisted of pedigree, phenotypes, and genotypes of Nelore cattle from the “Associação Nacional de Criadores e Pesquisadores” (ANCP). The association analyses were performed using the Weighted Single-Step Genome-Wide Association method; the regions, consisting of 10 consecutive SNP, which explained more than 0.5% of additive genetic variance, were considered as a significant association. A total of 3, 6, 7, 5, 10, 25, and 12 windows were associated with SC355, SC450, GL, AFC, ACP, HC30, and STAY, respectively. The results revealed genes with important functions for reproductive traits, such as fertility and precocity. Some genes were associated with more than one trait, among them *CAMK1D*, *TASP1*, *ACOXL*, *RAB11FIP5*, and *SFXN5*. Moreover, the genes were enriched in functional terms, like negative regulation of fat cell differentiation, fatty acid alpha-oxidation, and sphingolipids signaling pathway. The identification of the genes associated with the traits, as well as genes enriched in the terms and pathway mentioned above, should contribute to future biological validation studies and may be used as candidate genes in Nelore breeding programs.

## 1. Introduction

Greater inclusion of animal protein in human food has increased demand for beef production. Producers and researchers have been working, using the benefits of genetics, nutrition, and animal reproduction, on the search for a performance that is compatible with the growth of beef cattle production and that results in an increase in productivity [1,2].

Reproductive traits are economically relevant to beef cattle production systems [3]. Sexual precocity is especially important for *Bos taurus indicus* breeds that are commonly less precocious than *Bos taurus taurus* breeds [4]. Nevertheless, Zebu breeds have adaptive advantages in tropical production in comparison with Taurine breeds [5,6]. Reproductive inefficiency results in monetary losses caused by decreased production and detained reproduction, which impacts the costs and the sustainability of cattle production systems [7]. Therefore, reproductive traits must be considered in beef cattle production.

However, the functional reproductive traits selection is limited due to its small-moderate heritability [8,9] and result in a slow genetic gain. These traits are controlled by many genes and have relatively low to moderate heritability; that is, they are strongly influenced by environmental factors [10]. Thus, a small proportion of the phenotypic variance may be explained by genetic variance [9]. Furthermore, most reproductive traits are measured late in the life of the animals and are limited by sex, making selection and genetic gain difficult [11,12,13]. The response selection for reproductive traits is slower than for productive traits [14], which have intermediate to high heritability estimates. Thus, alternatives that improve the response to selection and consequently accelerate genetic gain are necessary to improve the reproductive performance of beef cattle herds. The genomic information has been included in the genetic evaluation, and even with the low heritability estimates of reproductive traits, a positive impact on fertility has been observed [8,15,16].

The selection focus on production traits and its antagonism with fertility traits in dairy cattle has caused a progressive decline in fertility. Thus, the inclusion of the reproductive traits in breeding programs and the use of genomic information benefit the identification of quantitative trait loci (QTL), and candidate genes associated with reproductive traits are highlighted [16]. García-Ruiz et al. [15] showed that the low heritability traits, such as fertility, are those that most benefit from the use of genomic information, increasing accuracy and selection differential and decreasing the generation interval.

Genomic studies have been widely used for beef cattle. Single nucleotide polymorphism (SNP) applied in genome-wide association studies (GWAS) can be used to identify possible associations between chromosomal regions and complex traits, such as reproductive traits. GWAS have been developed aiming to identify candidate genes and to understand the genetic mechanisms involved with the reproductive traits [17,18,19]. Thus, the use of more informative markers and the disposal of those that generate noise in the predictions could improve the accuracy of genomic predictions [20].

The SNP associated with genes that influence reproductive traits, once identified, can be incorporated into SNP panels to increase the accuracy of genomic predictions for fertility. Thus, contributing to the selection process [21] and making it possible, using single-step approaches, to evaluate genotyped young animals without their phenotypes [8] and increase the understanding of the mechanisms regulating the reproductive performance of beef cattle.

Genomic studies are the basis for accurate animal production [22]. Therefore, the aim of this study is to evaluate the association between SNP and scrotal circumference at 365 days of age (SC365) and at 450 days of age (SC450), gestation length (GL) as a calf trait, age at first calving (AFC), accumulated productivity (ACP), heifer early calving until 30 months (HC30) and stayability (STAY) traits, in order to identify candidate genes and biological pathways associated with reproductive traits in Nelore beef cattle.

## 2. Material and Methods

### 2.1. Data

The data set was composed of pedigree, phenotypes, and genotypes of Nelore beef cattle from the “Associação Nacional de Criadores e Pesquisadores” (ANCP). The ANCP animal breeding program aims to identify, select and offer to the beef market genetically superior animals for productive and reproductive traits. The pedigree used was composed of 140,199 Nelore animals born in the years 1934 to 2016, with 9.37 ± 5.64 mean generations known and 16.82% of missing parents rate (at least one unknown parent), and with an effective population size equal to 404. The phenotypic data set consisted of a total of 50,331 animals with observations for at least one of the SC365, SC450, GL, AFC, ACP, HC30, and STAY traits in Nelore beef cattle (Table 1).

The ACP index was calculated under the expression described in the study by Lôbo et al. [23]:

(1) $$\mathrm{ACP}=\frac{W_{210}\times n_{c}\times 365}{{\mathrm{ADC}}_{n}-550}$$

where W_210_ is the average body weight of weaned calves corrected for 210 days of age, n_c_ is the total number of calves produced, and ADC_n_ is the dam’s age at last calving. The 365 and 550 constants enable fertility expression on an annual basis.

HC30 and Stayability are bi-categorical traits. HC30 was defined attributing the value of 1 (success) for heifers first calving until 30 months of age, and 0 (failure) otherwise. For Stayability, cows that attained at least three calvings at 76 months of age had the phenotype categorized as “success”; otherwise, cows that had not had three calvings until this age had a phenotype described as “failure”.

The animals were raised on pasture, weaned between 6 and 8 months of age, and the reproductive management consisted of the mating season lasting from 60 to 120 days using artificial insemination or controlled natural mating. The contemporary groups (CG) were constituted by sex (when the trait was measured in both sexes), animals born on the same farm, year, and season and belonging to the same management group.

The genotypic data set was composed of 8652 animals (males and females). From these animals, a total of 960 bulls were genotyped with HD SNP panel (Illumina Bovine HD BeadChip) (Illumina, Inc., San Diego, CA, USA), 1000 animals were genotyped with a medium-density SNP panel (50k-Illumina BovineSNP50 BeadChip) (Illumina, Inc., San Diego, CA, USA), and 6692 animals were genotyped with a low-density SNP panel (12k-Clarifide Nelore 2.0) (Zoetis–Clarifide, San Diego, CA, USA). The animals genotyped with lower density were imputed to medium-density (50k) and subsequently to the HD panel using FImpute v.2.2 [24].

### 2.2. Quality Control

#### 2.2.1. Phenotype Data

The CG with a frequency of fewer than five animals was excluded. The connectability analysis between CG for each trait was performed using the AMC software [25], and disconnected CG was disregarded in the analyzes. The residual normality and variance homogeneity assumptions were performed, and observations with ±3.0 standard deviations were excluded for each trait. The number of observations after phenotype data editing is summarized in Table 1.

#### 2.2.2. Genotype Data

The SNP with a call rate less than 90% and minor allele frequency (MAF) less than 5% were excluded. Samples with a call rate of less than 90% were also not considered in analyses. Only autosomal SNPs were considered. After genomic data quality control, 460,838 SNPs and 8545 animals with observations for at least one trait were available.

### 2.3. Genome-Wide Association Analysis

The methodology used was the weighted single-step genome-wide association (WssGWAS); that is, modification of the BLUP method, with the numerator relationship matrix A^−1^ replaced by the genomic matrix H^−1^ [26]:

(2) $$H^{-1}=A^{-1}+\left[\begin{array}{cc} 0 & 0\\ 0 & G^{-1}-A_{22}^{-1} \end{array}\right]$$

where A is the numerator relationship matrix for all animals; $A_{22}$ is the numerator relationship matrix for genotyped animals, and G is the genomic relationship matrix. The G matrix was obtained according to Amin et al. [27] and Leuttenegger et al. [28]:

(3) $$G={\mathrm{ZDZ}}^{\prime }$$

where Z is the coefficient matrix adjusted for allele frequency; D is the matrix that contains the weights for all SNPs.

For the derivation of SNPs effects and weights, the animal effect was decomposed into genotyped animals ($a_{g}$) and not genotyped ($a_{n}$), as described by Wang et al. [29]. The animal effect of the genotyped animals is a function of the SNP effects:

(4) $$a_{g}=Z_{u}$$

where Z is a matrix relating genotypes of each loci and u is a vector of the SNP effects. The animal effect variance was calculated by:

(5) $$\mathrm{var}\left(a_{g}\right)=\mathrm{var}\left(Z_{u}\right)={\mathrm{ZD}Z^{\prime }\;\sigma }_{u}^{2}=G^{*}\sigma_{a}^{2}$$

where D is the diagonal matrix of weights for variances of SNP; $\sigma_{u}^{2}$ is the genetic additive variance captured by each SNP and $G^{*}$ is the weighted relationship genomic matrix, obtained by $G^{*}=\mathrm{Gq}$ where q is a weighting/normalizing factor. This factor was used according to Chen et al. [30], to ensure genomic evaluations unbiased; for this, the average diagonal in G to that of $A_{22}$.

Thus, the effect of SNPs was obtained following the equation taken from Wang et al. [29]:

(6) $$\hat{u}=\frac{\sigma_{u}^{2}}{\sigma_{a}^{2}}{{\mathrm{DZ}}^{\prime }G}^{*-1}{\hat{a}}_{g}={\mathrm{DZ}}^{\prime }{\left[{\mathrm{ZDZ}}^{\prime }\right]}^{-1}{\hat{a}}_{g}$$

The iterative process described by Wang et al. [29] in six steps was used: Step one, D=I; step two, to calculate ${\hat{a}}_{g}$ with WssGWAS; step three, to calculate the SNP effect; step four, to calculate the variance of each SNP, $d_{i}={\hat{u}}_{i}^{2}2p_{i}\left(1-p_{i}\right)$, where i is the ith SNP; step five, the normalized value of the SNP to keep the additive genetic variance constant and step six, to calculate the G matrix. A weighted process that recalculated the weights of SNPs consisted of repeating once steps 2 to 6 [29].

This process increases SNP weights explaining larger genetic variance and decreases SNP weights explaining small genetic variance. The percentage of genetic variance explained by the ith region was calculated as follows:

(7) $$\frac{\mathrm{Var}\left(a_{i}\right)}{\sigma_{i}^{2}}\times 100\%=\frac{\mathrm{Var}\left(\sum_{j=1}^{10}Z_{j}{\hat{u}}_{j}\right)}{\sigma_{a}^{2}}\times 100\%$$

where $a_{i}$ is the genetic value of the ith region that consists of 10 consecutive SNP, $\sigma_{a}^{2}$ is the total genetic variance, $Z_{j}$ is the vector of SNP content of the jth SNP for all individuals and ${\hat{u}}_{j}$ is the marker effect of the jth SNP within the ith region.

The analyzes for estimation of the genomic association were performed using software from the BLUPF90 program family [31,32]. The SC365, SC450, GL, AFC, and ACP traits were evaluated under a linear model using POSTGSF90 software, while for HC30 and STAY traits; a threshold model was applied using THRGIBBS1F90 software. The variance components and heritability estimates of traits are provided in the Supplementary Table S1. The general animal models used for SC365, SC450, AFC, ACP, STAY, and HC30 (8) and for GL (9) were:

(8) $$y_{t}=\mathrm{Xb}+Z_{1^{a}}+e$$

(9) $$y_{t}=\mathrm{Xb}+Z_{1^{a}}+Z_{2^{m}}+e$$

where $y_{t}$ is the vector of phenotypic observations for each trait, except for STAY and HC30, that is the threshold vector; b is the vector of fixed effects, that included CG; a is the vector of effects of the animals; m  is the vector of maternal effect, and e is the vector of residual effects; X, $Z_{1}$ and $Z_{2}$ are the incidence matrix for b, a, and m, respectively. The variances of a and e were calculated by:

(10) $$\left[\begin{array}{c} a\\ m\\ e \end{array}\right]=\left[\begin{array}{ccc} {H\sigma }_{a}^{2} & {H\sigma }_{\mathrm{am}} & 0\\ {H\sigma }_{\mathrm{am}} & {H\sigma }_{m}^{2} & 0\\ 0 & 0 & {I\sigma }_{e}^{2} \end{array}\right]$$

where $\sigma_{a}^{2}$ is the additive genetic variance; $\sigma_{m}^{2}$ is the maternal additive genetic variance; $\sigma_{e}^{2}$ is the residual variance; $\sigma_{\mathrm{am}}$ is the additive-maternal covariance and H is the matrix that combines the relationship and genomic information matrix, and I is the identity matrix.

### 2.4. Search for Associated Genes

The chromosome regions, consisting of windows with 10 consecutive SNPs that explained more than 0.5% of additive genetic variance were considered as associated with the studied trait [33], and then the investigation of the genes that this region contained was carried out with Ensembl Genome Browser (http://www.ensembl.org/index.html accessed on 18 October 2018) using the Ensembl gene 94 version of the gene model and UMD3.1.1 bovine genome assembly as reference. The enrichment of genes, as well as their classification according to biological function and identification of metabolic pathways, was performed using the “Database for Annotation, Visualization and Integrated Discovery (DAVID) v. 6.8” (http://david.abcc.ncifcrf.gov/ accessed on 14 November 2018) for each trait, with a *p*-value significance criterion of 0.05.

## 3. Results and Discussion

**Figure 1:**
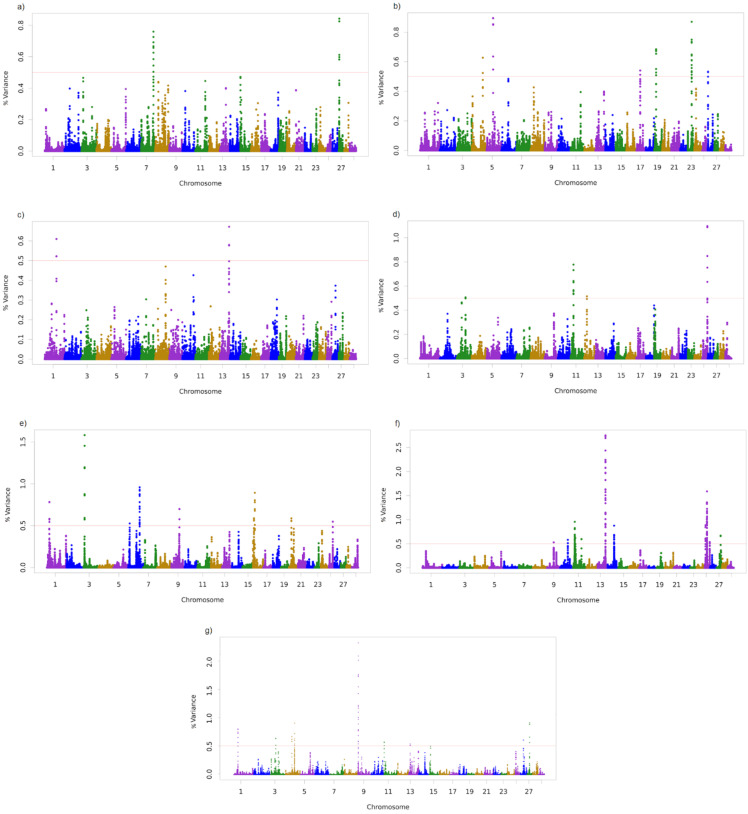
Additive genetic variance explained by windows of 10 adjacent SNP distributed by chromosomes for (**a**) Scrotal circumference at 365 days of age (SC365), (**b**) Scrotal circumference at 450 days of age (SC450), (**c**) Gestation length (GL), as a calf trait, (**d**) Age at first calving (AFC), (**e**) Accumulated productivity (ACP), (**f**) Early calving until 30 months (HC30), and (**g**) Stayability (STAY).

The associated regions with the traits studied are shown in Figure 1 and presented in Table 2. A total of 3, 6, 2, 5, 10, 25, and 12 windows with 10 SNP were associated with SC355, SC450, GL, AFC, ACP, HC30, and STAY, respectively.

The genes found in the significant regions for each trait are presented in Supplementary Table S2. In the regions associated with SC365 (Table 2), no gene was found. A total of five known genes and three unknown genes are associated with SC450, and in the enrichment analysis, no term was significantly enriched (*p* > 0.05). A single known gene (*CDH22*) was found in GL-associated regions (Table S2), and in the enrichment analysis, no term was significantly enriched (*p* > 0.05).

A total of 231 genes were found in the AFC-associated regions (Table S2), with 178 known and 53 unknown genes. The enrichment analysis (*p* ≤ 0.05) for AFC-associated genes in six terms (Table S3), including mitochondrial translational initiation and elongation. In these pathways, the *mitochondrial ribosomal protein S5* (*MRPS5*) and *mitochondrial ribosomal protein S9* (*MRPS9*) genes have been enriched and have already been associated with feed intake [34]. Basarab et al. [35] showed that animals with positive feed intake consumed more feed and retained more energy. The AFC has a negative genetic correlation with weaning and yearling weight gain (−0.20 and −0.24, respectively), indicating that the selection for greater weight gain results in an AFC decrease [36]. In Brazil, the Nelore is selected for weight gain. This can impact the sexual precocity of the animals, resulting in lower AFC [36]; in addition, it is noteworthy that the selection for weight gain can also increase the weight of adult females [37,38], which may not be interesting depending on the production system.

A total of 244 genes were found in the ACP-associated regions (Table S2), of which 156 are known genes, and 88 are unknown. A total of 22 terms (Table S4) were significantly enriched (*p* ≤ 0.05), being related to immune system processes and body growth.

The *neuropeptide Y receptor Y1* (*NPY1R*) gene was enriched in the regulation of multicellular organism growth term. The *NPY1R* has been found in association with Angus heifers’ fertility by Neupane et al. [39], and the neuropeptide Y associated with maternal behavior dependent on nutritional status in mice [40]. Adding to this, and considering that ACP indicates the abilities of the female to calve at a young age, to maintain the regularity of calving, and to wean heavy calves [41], the maternal behavior can influence the weaning weight of animals, and cow fertility can influence not only in AFC but also to maintain a shorter calving interval, which reflects the occurrence of regular calving, evidencing the importance of the *NPY1R* gene for a cow’s ACP throughout life.

In the regions associated with HC30, 580 genes were found (Table S2), of which 434 are known and 146 are unknown. Analyzing the total of genes, 22 terms (Table S5) were significantly enriched (*p* ≤ 0.05), among them fatty acid alpha-oxidation, negative regulation of fat cell differentiation, and innate immune response were highlighted.

The hydroxyacid oxidase (glycolate oxidase) 1 (HAO1), phytanoyl-CoA 2-hydroxylase (PHYH), and phytanoyl-CoA dioxygenase peroxisomal-like (LOC100300115) genes were enriched into the fatty acid alpha-oxidation term, that is the pathway that degrades 3-methyl branched fatty acids, which are not degraded by the normal degradation pathway of fatty acids (peroxisomal beta-oxidation pathway) [42]. The fatty acids metabolism is associated with successful pregnancy [18].

Into the negative regulation of fat cell differentiation term, the E2F transcription factor 1 (E2F1), GATA binding protein 3 (GATA3), additional sex combs like 1, transcriptional regulator (ASXL1), and tribbles pseudokinase 3 (TRIB3) genes were enriched. This term involves processes that inhibit the differentiation of adipocytes [42]. Considering the association of the two terms mentioned above with HC30, the results in the present study suggest that the favorable selection of alleles that inhibit degradation of 3-methyl branched fatty acids and which favor differentiation of adipocytes can result in higher fat deposition in these animals, which would be sexually precocious, i.e., with more success for HC30.

The biological process of innate immune response was significantly enriched (*p* ≤ 0.05) associated with the HC30 trait. Melo et al. [18] identified an association between the number of calves at 53 months of age trait and the immune processes pathway. The phenotypic correlations were reported by Banos et al. [43] between immune traits with reproductive performance traits; in especial, the authors reported that a higher concentration of CD8+ cells was associated with longer calving intervals. Thus, the immune system can interfere not only in the HC30 trait but also in cow longevity since the longer calving interval results in a lower stay in the herd.

A total of 52 genes were found in the STAY-associated regions (Table S2), of which 37 are known genes, and 15 are unknown genes. The analysis of the biological functions of the genes enriched six terms (*p* ≤ 0.05) (Table S6), among them, the sphingolipid signaling pathway. The lipid pathways are associated with the reproductive traits, such as the Nelore heifer reconception trait, indicating that cows with greater capacity to accumulate fat have better rebreeding performance [18] and consequently greater STAY.

The sphingolipid signaling and its metabolic products have a signaling function in several other pathways, such as the metabolic sphingosine-1-phosphate (S1P) that acts as a growth and survival factor and the ceramide (Cer) that activates the apoptotic pathways [44]. The relation of this pathway to the activation of growth factor interferes in the sexual precocity of the heifers, which can take less time to reach the necessary weight for reproductive maturity, thus decreasing AFC and increasing the cow’s STAY. By activating survival factors, this pathway could also be associated with the longevity of cows in the herd, and thus, in STAY.

The *phospholipase C beta 1* (*PLCB1*), enriched in the sphingolipid signaling pathway, and *phospholipase C beta 4* (*PLCB4*) genes, both located in BTA 13, were associated with STAY. Early embryonic development in mammals depends on maternal miRNA stocks prior to the initiation of zygote regulation; this miRNA involvement has been described in rats [45] and bovine animals [46]. The *PLCB1* is a target gene of the miR-205, and this miRNA was differentially expressed during preimplantation embryonic development, and both *PLCB1* and miR-205 have a reciprocal expression pattern [46]. The *PLCB1* and *PLCB2* genes are targets of the miR-301b, which is associated with ovarian follicle development in cattle [47].

The *jagged 1* (*JAG1*) and *mitochondrial ribosomal protein L33* (*MRPL33*) genes, both located in BTA 13, were found associated with STAY. Although these genes have not been enriched in biological pathways, they have important reproductive functions. The *JAG1* gene has a signaling function during embryogenesis [48], and the *MRPL33* gene was cited by Melo [49] for being in association with heifer rebreeding in Nelore cattle. This fact reinforces the association between *MRPL33* and STAY found in our work because the stayability of a cow in the herd is influenced by its ability for reconception even when it is a heifer.

Many uncharacterized genes were found associated with the traits. These genes do not yet have an identified ortholog, which means that they do not have a homologous gene that has the same function in different organisms; therefore, in the enrichment analyzes, these genes were not found. All the biological processes, cell components, molecular functions, and pathways that were significantly enriched (*p* ≤ 0.05) in the gene ontology analysis are presented in Supplementary Tables S3–S6.

None of the genes found in the associated regions with each trait studied were associated with all the traits. The associated genes with SC450, GL, and ACP did not coincide with any gene associated with the other traits. According to Kluska et al. [50], using part of the database of this manuscript, the SC450 presented genetic correlation with AFC, HC30, and STAY equal to −0.29 ± 0.06, 0.52 (0.31–0.74) and 0.35 (0.20–0.50), respectively. This indicates that selection for SC can decrease AFC [51] and increase HC30 and STAY; therefore, it is an indicator of the sexual precocity of Nelore heifers. Grossi et al. [41] found a negative genetic correlation between ACP and AFC (−0.33 ± 0.04) in Nelore dams, i.e., the gains in ACP are related to a lower AFC. The genetic correlation between these traits, related by Kluska et al. [50] and Grossi et al. [41] indicates that these traits are controlled by common genes, reinforcing the GWAS results of this paper that found some common genes associated with the traits.

The common genes associated with the reproductive traits are presented in Table S7. A single common and known gene is associated with AFC and STAY; 3 common genes are in association with AFC and HC30 (2 known genes); 31 genes common to HC30 and STAY (22 known genes).

Different studies reported that AFC and STAY present negative genetic correlation (−0.64 (−0.83–−0.44); −0.69 and −0.32 (−0.40–−0.25) [9,50,52]; thus, the decrease in AFC would lead to an increase in STAY. The *acyl-CoA oxidase like* (*ACOXL*) gene located in the BTA 11 was found in the regions associated with AFC and STAY, so the selection targeted to this gene would allow a possible gain for both traits. *ACOXL* is a primary rate-limiting enzyme in peroxisomal fatty acids β-oxidation [53,54] and has been found in association with pregnancy outcome (pregnant or not pregnant) using fixed-time artificial insemination in Brahman heifers [55].

The *RAB11 Family Interacting Protein 5* (*RAB11FIP5*) and *Sideroflexin 5* (*SFXN5*) genes, both located in BTA 11, were associated with AFC and HC30. The *RAB11FIP5* and *SFXN5* genes have already been described as associated with shear force [56], a method used to evaluate the tenderness of meat. There is a high degree of interdependence of puberty traits and growth and body composition [57]. Pacheco et al. [58] reported that subcutaneous fat thickness is favored when younger animals are slaughtered. Still, part of a chromosomal region associated with the occurrence of early calving is also described when meat tenderness in Nelore cattle is investigated [17,59]. In view of this, the *RAB11FIP5* and *SFXN5* genes seem to influence sexual precocity in cattle, justifying the association of these genes with AFC and HC30 traits identified in the present study.

The gene *calcium/calmodulin dependent protein kinase ID* (*CAMK1D*) was found associated with HC30 and STAY. This gene is located in BTA 13, and its association with heifer rebreeding and age at first calving was found by Costa et al. [60] in a GWAS study of Nelore dams. In addition, Melo et al. [19] reported that this gene might contribute to sexual precocity in Nelore cattle.

*Threonine aspartase 1* (*TASP1*) gene, also associated with HC30 and STAY, generates alpha and beta subunits, forming the active alpha2-beta2, necessary to cleave the protein used for the maintenance of *HOX* gene expression [61]. When the *HOX* gene has its expression altered, it interferes with uterine development, preventing the implantation of the embryo and causing infertility in women [62]. In bovine, the *HOX* expression profile suggests a role during early developmental events, including oocyte maturation, the maternal to embryonic transition, and the first differentiation events [63]. In the same study, the comparison between two bovine and mouse species indicated that *HOX* expression at initial stages is partly conserved among mammals. Thus, the idea of the influence of the *TASP1* gene on cow fertility is reinforced and justifies its association with STAY and HC30 traits.

## 4. Conclusions

The results in the present study provide a better understanding of the genes associated with reproductive traits studied in Nelore cattle. Many genes found in the associated windows appear to be related to more than one trait, indicating that these genes have a pleiotropic effect and are important for reproductive efficiency. Among these common genes, the *CAMK1D* and *TASP1* genes were associated with HC30 and STAY; the *ACOXL* gene that appeared to be associated with AFC and STAY, and *RAB11FIP5* and *SFXN5* genes associated with AFC and HC30 should be highlighted due to their relationship with reproductive efficiency.

Many pathways and terms were associated with the reproductive traits in Nelore cattle, but the fatty acid alpha-oxidation and negative regulation of fat cell differentiation terms stand out for being related with HC30, while the sphingolipid signaling pathway was highlighted as being related to STAY. The identification of the genes associated with the traits, as well as genes enriched in the terms and pathway mentioned above, should contribute to future biological validation studies and may be used as candidate genes in Nelore breeding programs.

## Figures and Tables

**Table 1:** Number of observations (*n*), mean and respective standard deviation (SD), minimum, maximum, and number of contemporary groups (CG) for scrotal circumference at 365 days of age (SC365), scrotal circumference at 450 days of age (SC450), gestation length (GL) as a calf trait, age at first calving (AFC), accumulated productivity (ACP), heifer early calving until 30 months (HC30), and stayability (STAY).

Trait	*n*	Mean *	SD	Minimum	Maximum	CG
SC365 (cm)	15,054	21.56	2.40	14.40	29.10	426
SC450 (cm)	13,694	25.01	3.23	16.00	35.40	381
GL (days)	16,252	296.80	6.07	278.00	314.00	202
AFC (months)	15,384	35.24	5.91	21.00	49.00	237
ACP (kg)	6205	150.70	31.12	63.00	249.00	150
HC30	3236	0.30	-	0	1.00	97
STAY	12,981	0.42	-	0	1.00	162

* For HC30 and STAY, the mean represents the “success” proportion for each trait.

**Table 2:** Genomics regions associated with a scrotal circumference at 365 days of age (SC365) and 450 of age (SC450), gestation length (GL) as calf trait, age at first calving (AFC), accumulated productivity (ACP), heifer early calving until 30 months (HC30) and stayability (STAY) traits in Nelore cattle and percentage of additive genetic variance.

Chromosome	Position(bp)	% Additive Genetic Variance
Scrotal circumference at 365 days of age		
BTA7	99,797,785–99,807,157	0.7598
BTA27	3,459,974–3,476,332	0.5827
BTA27	3,763,511–3,783,637	0.8425
Scrotal circumference at 450 days of age		
BTA4	93,491,117–93,502,503	1.7190
BTA5	54,114,064–5,415,865	2.3197
BTA17	30,807,597–30,828,891	0.5404
BTA19	16,748,888–16,777,564	0.6842
BTA23	30,929,417–30,952,562	1.2410
BTA26	489,143–4,913,438	2.7796
Gestation length		
BTA1	89,275,294–89,301,259	0.6100
BTA13	75,605,755–75,651,541	0.6721
Age at first calving		
BTA3	71,141,852–71,202,433	0.5065
BTA11	21,246–21,281,912	0.7776
BTA11	21,287,816–21,311,113	0.7324
BTA12	19,870,944–19,879,683	0.5148
BTA25	40,542,599–40,557,377	1.0960
Accumulated productivity		
BTA1	2,054,452–20,570,066	0.7828
BTA3	7,752,656–7,785,494	1.5813
BTA6	1,442,287–14,434,633	0.5264
BTA6	94,344,402–94,375,956	0.5681
BTA6	94,388,672–94,426,934	0.9580
BTA9	67,670,626–67,696,355	0.6988
BTA16	9,824,597–9,861,772	0.5885
BTA16	1,706,924–17,085,278	0.8929
BTA20	19,183,661–19,199,006	0.5869
BTA25	31,194,971–31,217,552	0.5478
Heifer early calving until 30 months		
BTA9	56,459,368–56,477,584	0.5289
BTA10	63,914,118–63,931,413	0.5813
BTA11	11,320,434–11,348,287	0.5008
BTA11	11,355,768–11,368,368	0.6445
BTA11	11,374,317–11,381,414	0.9560
BTA11	11,410,105–11,424,583	0.5888
BTA11	11439886–11,463,529	0.5572
BTA11	11496633–11,536,209	0.6600
BTA11	16,578,809–16,627,592	0.6191
BTA11	67,791,729–67,805,289	0.5965
BTA13	64,147,929–64,202,735	0.5643
BTA13	64,212,843–64,261,331	1.5723
BTA13	6,426,382–64,340,236	2.7497
BTA13	64,358,933–64,432,139	1.1148
BTA13	64,450,162–64,500,844	0.5546
BTA14	51,334,426–51,347,339	0.5336
BTA14	51,399,505–5,142,635	0.5006
BTA14	51,586,769–51,622,257	0.8759
BTA25	7,204,736–722,268	0.8833
BTA25	17,664,012–17,694,691	1.3587
BTA25	18,619,922–18,639,119	0.7122
BTA25	18,672,747–18,714,923	1.5879
BTA25	18,946,238–18,966,876	1.3475
BTA25	40,714,999–40,732,777	0.5351
BTA27	35,208,391–35,220,792	0.6705
Stayability		
BTA1	33,285,504–33,305,018	0.8321
BTA3	43,009,131–43,028,743	0.6630
BTA4	55,984,897–56,013,042	0.6909
BTA4	79,187,125–79,206,524	0.9377
BTA4	79,221,965–7,930,004	0.6522
BTA9	4,370,684–439,556	2.4011
BTA9	4,448,707–4,477,071	1.2592
BTA11	1,424,088–1,433,201	0.5943
BTA13	1,339,928–13,434,427	0.5580
BTA15	15,283,071–15,318,647	0.5176
BTA26	25,583,443–25,591,332	0.6311
BTA27	22,208,624–22,221,777	0.9445

## Data Availability

The data that support the findings of this study are available upon reasonable request contacting Dr. Raysildo Barbosa Lôbo, raysildo@ancp.org.br. Tel.: (+55) 16 3602 3252, Universidade de São Paulo, Faculdade de Medicina de Ribeirão Preto, Departamento de Genética. Av. Bandeirantes 3900, Monte Alegre, 14040030 Ribeirão Preto, SP, Brasil.

## Supplementary Materials

The following are available online at www.mdpi.com/xxx/s1.Table S1: Estimations of genetic variance ($\sigma_{a}^{2}$), genetic variance of maternal effect ($\sigma_{m}^{2}$), genetic standard deviation ($\sigma_{a}$), genetic standard deviation of maternal effect ($\sigma_{m}$), residual variance ($\sigma_{e}^{2})$, residual standard deviation ($\sigma_{e})$, heritability ($h_{a}^{2})$, and heritability of maternal effect ($h_{m}^{2})\;$ for scrotal circumference at 365 days of age (SC365), scrotal circumference at 450 days of age (SC450), gestation length (GL), as a calf trait, age at first calving (AFC), accumulated productivity (ACP), early calving until 30 months (HC30), and stayability (STAY). Table S2: Genes in significant genomics regions associated with reproductive traits in Nelore cattle. Table S3: Enriched GO terms and KEGG pathways significantly enriched (*p* ≤ 0.05) from DAVID software for age at first calving. Table S4: Enriched GO terms and KEGG pathways significantly enriched (*p* ≤ 0.05) from DAVID software for accumulated productivity. Table S5: Enriched GO terms and KEGG pathways significantly enriched (*p* ≤ 0.05) from DAVID software for heifer early calving until 30 months. Table S6: Enriched GO terms and KEGG pathways significantly enriched (*p* ≤ 0.05) from DAVID software for stayability. Table S7: Common gene list associated with de with reproductive traits in Nelore cattle
