# Supplementary material for: Genome-Wide Association Study Provides Insights into Important Genes for Reproductive Traits in Nelore Cattle
Source: Animals (Basel). 2021 May 13;11(5):1386. doi: 10.3390/ani11051386 (PMC8152989; doi:10.3390/ani11051386)
Supplement: Supplementary file 1 [file animals-11-01386-s001.zip › animals-1179537-supplementary.pdf]

animals

MDPI

Supplementary Materials: Genome-Wide Association Study Provides Insights into Important Genes for Reproductive Traits in Nelore Cattle

Ana Paula Sbardella, Rafael Nakamura Watanabe, Rebeka Magalhães da Costa, Priscila Arrigucci Bernardes, Larissa Graciano Braga, Fernando Sebastián Baldi Rey, Raysildo Barbosa Lôbo and Danísio Prado Munari

Table S1. Estimations of genetic variance (σ_a^2), genetic variance of maternal effect (σ_m^2), genetic standard deviation (σ_a), genetic standard deviation of maternal effect (σ_m), residual variance (σ_e^2), residual standard deviation (σ_e), heritability (h_a^2) and heritability of maternal effect (h_m^2) for scrotal circumference at 365 days of age (SC365), scrotal circumference at 450 days of age (SC450), gestation length (GL), as a calf trait, age at first calving (AFC), accumulated productivity (ACP), early calving until 30 months (HC30) and stayability (STAY).

Variance components and heritability estimates							
**Parameters**	**SC355**	**SC450**	**GL**	**AFC**	**ACP**	**HC30**	**STAY**
σ_a^2	1.2752	2.7342	17.149	3.1291	117.840	1.847	0.4163
σ_m^2	-	-	1.549	-	-	-	-
σ_a	0.10086	0.20396	1.5803	0.347	19.634	0.402	5.567E-02
σ_m	-	-	0.45191	-	-	-	-
σ_e^2	1.9038	2.8895	14.875	16.272	542.710	1.002	1.009
σ_e	7.63E-02	0.1496	0.90241	0.32625	18.649	3.865E-02	1.801E-02
h_a^2	0.401	0.486	0.511	0.161	0.178	0.648	0.292
h_m^2	-	0.046	-	-	-	-	-

Table S2. Genes in significant genomics regions associated with reproductive traits in Nelore cattle.

Trait
**Chromosome: Position(pb)**<br>**Gene**
**Scrotal circumference at 450 days of age**
19:16748888-16777564<br>ASIC2
26:489143-4913438<br>UBE2D1, CISD1, IPMK, ZWINT, ENSBTAG00000023971, ENSBTAG00000048299, ENSBTAG00000047692
**Gestation length, as a calf trait**
13:75605755-75651541<br>CDH22
**Age at first calving**
11:21246-21281912<br>ENSBTAG00000038257, ZC3H6, ZC3H8, FBLN7, TMEM87B, ENSBTAG00000044282, RF00401, MERTK, RF00156, ANAPC1, RF00026, ENSBTAG00000023163, BCL2L11, ACOXL, BUB1, TPC3, ENSBTAG00000045538, NPHP1, MALL, MAL, ENSBTAG00000034657, MRPS5, ZNF514, ZNF2, ENSBTAG00000047503, PROM2, ENSBTAG00000017955, FAHD2A, GPAT2, ADRA2B, ASTL, DUSP2, STARD7, TMEM127, CIAO1, SNRNP200, ENSBTAG00000047659, ITPRIPL1, NCAPH, NEURL3, ARID5A, KANSL3, FER1L5, LMAN2L, CNNM4, CNNM3, ANKRD23, ANKRD39, SEMA4C, FAM178B, COX5B, ACTR1B, ZAP70, TMEM131, VWA3B, CNGA3, INPP4A, COA5, UNC50, MGAT4A, RF00425, TSGA10, C2orf15, LIPT1, MITD1, MRPL30, LYG2, TXNDC9, EIF5B, REV1, ENSBTAG00000012449, LONRF2, CHST10, NMS, PDCL3, PTPN13, ENSBTAG00000016794, NPAS2, RPL31, TBC1D8, CNOT11, RF00578, RNF149, CREG2, RFX8, MAP4K4, IL1R2, RF00594, IL1R1, RF00001, ENSBTAG00000046579, IL1RL2, IL1RL1, IL18R1, IL18RAP, SLC9A4, SLC9A2, MFSD9, TMEM182, MRPS9, GPR45, TGFBRAP1, C11H2orf49, FHL2, TACR1, POLE4, HK2, ENSBTAG00000044983, SEMA4F, M1AP, DOK1, LOXL3, HTRA2, AUP1, DQX1, TLX2, PCGF1, LBX2, CCDC142,

	MRPL53, MOGS, WBP1, INO80B, RTKN, WDR54, C11H2orf81, MGC152281, DCTN1, SLC4A5, ENSBTAG00000010072, MTHFD2, MOB1A, BOLA3, TET3, DGUOK, ACTG2, STAMBP, C2orf78, DUSP11, bta-mir-2295, TPRKB, NAT8, ALMS1, EGR4, FBXO41, CCT7, PRADC1, SMYD5, NOTO, ENSBTAG00000008035, RF00100, RAB11FIP5, bta-mir-2294, SFXN5, EMX1, SPR, EXOC6B, ENSBTAG00000046679, ENSBTAG00000039263, CYP26B1, DYSF, ZNF638, PAIP2B, NAGK, TEX261, ANKRD53, ATP6V1B1, VAX2, CD207, CLEC4F, FIGLA, ADD2, TGFA, FAM136A, XDH, SRD5A2, MEMO1, ENSBTAG00000033887, DPY30, SPAST, SLC30A6, NLRC4, YIPF4, BIRC6, TTC27, ENSBTAG00000037357, LTBP1, RASGRP3, ENSBTAG00000045057, FAM98A, RF00066, RF00015, CRIM1, FEZ2, VIT, STRN, HEATR5B, GPATCH11, EIF2AK2, SULT6B1, CEBPZ, NDUFAF7, PRKD3, QPCT, CDC42EP3, RMDN2, CYP1B1, ATL2, RF01161, HNRNPLL, ENSBTAG00000044545, ENSBTAG00000026809, GALM, SRSF7, ENSBTAG00000012984, GEMIN6, DHX57, MORN2, ARHGEF33
11:21287816-21311113	ARHGEF33
**Accumulated productivity**	
1:2054452-20570066	PAXBP1, SYNJ1, SYNJ1, CFAP298, EVA1C, URB1, MRAP, MIS18A, HUNK, SCAF4, SOD1, TIAM1, TIAM1, bta-mir-2284i, bta-mir-2284x, ENSBTAG00000004530, KRTAP11-1, KRTAP7-1, KRTAP8-1, ENSBTAG00000044154, RF00026, KRTAP13-1, ENSBTAG00000037933, KRTAP27-1, KRTAP24-1, CLDN8, CLDN17, ENSBTAG00000030124, GRIK1, GRIK1, ENSBTAG00000045873, RF00026, BACH1, MAP3K7CL, CCT8, USP16, RWDD2B, RWDD2B, LTN1, ENSBTAG00000038433, N6AMT1, RF00026, ENSBTAG00000040155, RF00432, ADAMTS5, ADAMTS1, RF00026, ENSBTAG00000005742, RF00026, APP, RF00026, GABPA, ATP5PF, ATP5PF, JAM2, MRPL39, bta-mir-155, RF00026, RF00026, ENSBTAG00000038900, ENSBTAG00000048165, RF00100, NCAM2, RF00001, ENSBTAG00000039704, RF00026, RF00001, RF00001, TMPRSS15, TMPRSS15, CHODL, C1H21orf91, RF00026, ENSBTAG00000047943, BTG3, CXADR, RF00100, ENSBTAG00000040418, bta-mir-125b-2, MIRLET7C, bta-mir-99a, ENSBTAG00000047183, RF00026
3:7752656-7785494	ATF6
6:1442287-14434633	MARCH1, TMA16, TKTL2, NPY5R, NPY1R, NAF1, RF00026, BBS7, CCNA2, EXOSC9, ANXA5, RF00012, RF00026, QRFPR, NDNF, PRDM5, RF00026, RF00001, ENSBTAG00000037426, ENSBTAG00000047900, ENSBTAG00000024874, ENSBTAG00000035129, ENSBTAG00000035129, MAD2L1, ENSBTAG00000035764, ENSBTAG00000035764, ENSBTAG00000040228, ENSBTAG00000037311, PDE5A, FABP2, C6H4orf3, USP53, RF00001, MYOZ2, SYNPO2, SEC24D, METTL14, PRSS12, RF00399, ENSBTAG00000010010, NDST3, NDST3, ENSBTAG00000034844, RF00001, RF00001, RF00001, RF00001, RF00001, RF00001, RF00001, RF00001, ENSBTAG00000034849, TRAM1L1, ENSBTAG00000000119, ENSBTAG00000009280, NDST4, UGT8, ENSBTAG00000011299, ARSJ, CAMK2D, CAMK2D, ENSBTAG00000002392, ENSBTAG00000002392, ENSBTAG00000002392, LARP7, bta-mir-302b, bta-mir-302c, bta-mir-302a, bta-mir-302d, bta-mir-367, ENSBTAG00000026936, ENSBTAG00000013198, NEUROG2, ALPK1, ALPK1, ALPK1, TIFA, AP1AR
6:94344402-94375956	MRPL1
6:94388672-94426934	MRPL1
16:1706924-17085278	SOX13, ETNK2, REN, KISS1, GOLT1A, PLEKHA6, PLEKHA6, PLEKHA6, PPP1R15B, PIK3C2B, PIK3C2B, RF00334, MDM4, ENSBTAG00000026068, ENSBTAG00000034611, LRRN2, RF01169, NFASC, CNTN2, TMEM81, RBBP5, DSTYK, TMCC2, TMCC2, NUAK2, ENSBTAG00000047727, KLHDC8A, LEMD1, bta-mir-135b, CDK18, MFSD4A, ELK4, ELK4, SLC45A3, NUCKS1, RAB29, SLC41A1, PM20D1, SLC26A9, RAB7B, RHEX, AVPR1B, FAM72A, SRGAP2, IKBKE, RASSF5, EIF2D, DYRK3, MAPKAPK2, IL10, IL19, IL19, IL20, PIGR, PIGR, PIGR, FCAMR, FCMR, IL24, ENSBTAG00000046946, C16H1orf116, YOD1, PFKFB2, PFKFB2, C4BPB, C4BPA, C4BPA, ENSBTAG00000039196, ENSBTAG00000047621, CD55, CD55, CD55, ENSBTAG00000045783, CR2, CR2, ENSBTAG00000023177, ENSBTAG00000024647, ENSBTAG00000047228, ENSBTAG00000040409, CFH, ENSBTAG00000047966, KCNT2, ENSBTAG00000044933, ENSBTAG00000025113, ENSBTAG00000020953, RF00019, ENSBTAG00000014862, RF00004, ENSBTAG00000012765, CDC73, ENSBTAG00000047703, ENSBTAG00000046060, B3GALT2, GLRX2, TROVE2,

UCHL5, ENSBTAG00000044446, RGS2, RGS13, RGS1, RGS21, RGS18, RF00001, RF00156, BRINP3, ENSBTAG00000045416, RF00001, RF00026
**Heifer early calving until 30 months**
11:11320434-11348287<br>RAB11FIP5
11:11355768-11368368<br>RAB11FIP5, SFXN5
11:11374317-11381414<br>SFXN5
11:11410105-11424583<br>SFXN5
11:11439886-11463529<br>SFXN5
11:11496633-11536209<br>EMX1
11:67791729-67805289<br>ENSBTAG00000047822
13:64212843-64261331<br>RF00045, ASIP, AHCY
13:6426382-64340236<br>SPTLC3, RF00273, ISM1, TASP1, TASP1, ESF1, NDUFAF5, SEL1L2, FLRT3, MACROD2, KIF16B, ENSBTAG00000032609, SNRPB2, OTOR, NANP, ENSBTAG00000023169, ENSBTAG00000004354, ENSBTAG00000022922, ENSBTAG00000022922, ENSBTAG00000040046, CCDC3, CAMK1D, RF00619, CDC123, NUDT5, SEC61A2, DHTKD1, UPF2, RF00012, PROSER2, ECHDC3, USP6NL, CELF2, RF00026, ENSBTAG00000045874, ENSBTAG00000048047, ENSBTAG00000048047, GATA3, TAF3, ATP5F1C, KIN, ITIH2, ENSBTAG00000037516, ITIH5, SFMBT2, RF00026, RF00026, PRKCQ, PFKFB3, RBM17, IL2RA, IL2RA, ENSBTAG00000006078, FBH1, ENSBTAG00000036222, ENSBTAG00000036222, ENSBTAG00000017012, ENSBTAG00000022931, ENSBTAG00000022931, ENSBTAG00000034145, ANKRD26, YME1L1, MASTL, ACBD5, ACBD5, ABI1, PDSS1, APBB1IP, CCNY, CREM, CREM, CUL2, PARD3, NRP1, ITGB1, ITGB1, CCDC7, ARL5B, ENSBTAG00000047430, ENSBTAG00000037969, MALRD1, ENSBTAG00000044751, RF00026, PLXDC2, NEBL, ENSBTAG00000003937, SKIDA1, MLLT10, DNAJC1, DNAJC1, ENSBTAG00000044566, ENSBTAG00000025968, BMI1, SPAG6, COMMD3, PIP4K2A, RF00020, RF00026, RF00019, ARMC3, ARMC3, MSRB2, PTF1A, ENSBTAG00000044137, ENSBTAG00000044137, OTUD1, RF00100, RF00425, ENSBTAG00000040232, KIAA1217, ARHGAP21, ENSBTAG00000044011, ENKUR, RF00026, THNSL1, GPR158, MYO3A, GAD2, GJD4, FZD8, ENSBTAG00000047440, ENSBTAG00000044876, RF00619, OPTN, MCM10, ENSBTAG00000047869, PHYH, SEPHS1, BEND7, PRPF18, FRMD4A, FAM107B, CDNF, HSPA14, ENSBTAG00000045609, RF00099, SUV39H2, DCLRE1C, MEIG1, OLAH, ACBD7, RPP38, NMT2, NMT2, FAM171A1, RF00026, ITGA8, MINDY3, PTER, C1QL3, RSU1, CUBN, TRDMT1, VIM, ST8SIA6, HACD1, STAM, RF00153, TMEM236, MRC1, SLC39A12, CACNB2, NSUN6, EPC1, ENSBTAG00000043839, KIF5B, RF00026, ARHGAP12, ZEB1, RF01233, ZNF438, ENSBTAG00000027444, ENSBTAG00000027444, ENSBTAG00000027444, ENSBTAG00000046981, ENSBTAG00000046352, JCAD, MTPAP, MAP3K8, LYZL1, BAMBI, WAC, MPP7, ARMC4, MKX, RAB18, ENSBTAG00000048079, PCSK2, PCSK2, ENSBTAG00000006043, BFSP1, DSTN, RRBP1, BANF2, SNX5, RF00567, MGME1, OVOL2, OVOL2, KAT14, ZNF133, ZNF133, DZANK1, POLR3F, RBBP9, SEC23B, DTD1, ENSBTAG00000012276, RF00001, SCP2D1, SLC24A3, ENSBTAG00000032893, RIN2, bta-mir-2305, NAA20, CRNKL1, CFAP61, INSM1, RALGAPA2, RF00156, KIZ, XRN2, NKX2-4, NKX2-2, ENSBTAG00000035643, PAX1, FOXA2, RF00026, THBD, CD93, NXT1, GZF1, NAPB, CSTL1, CST11, CST11, CST11, MGC133636, ENSBTAG00000027420, CST8, CST8, ENSBTAG00000015949, ENSBTAG00000047137, ENSBTAG00000032687, CST3, SYNDIG1, CST7, CST7, APMAP, ENTPD6, ACSS1, VSX1, PYGB, ABHD12, ANKRD16, ENSBTAG00000035572, ENSBTAG00000002446, GDI2, FAM208B, RF00322, ASB13, ENSBTAG00000027416, CALML5, NET1, UCN3, TUBAL3, TUBAL3, ENSBTAG00000037509, ENSBTAG00000039722, ENSBTAG00000039643, ENSBTAG00000017938, ENSBTAG00000027419, AKR1C3, AKR1C4, AKR1C4, ENSBTAG00000039289, ENSBTAG00000021368,

ENSBTAG00000022570, 20ALPHA-HSD, AKR1E2, ENSBTAG00000017191, KLF6, ENSBTAG00000046128, PITRM1, PFKP, RF00402, ADARB2, ENSBTAG00000039356, ENSBTAG00000039356, ENSBTAG00000039356, ENSBTAG00000037833, WDR37, IDI1, GTPBP4, RF00026, LARP4B, DIP2C, RF00026, RF00089, ZMYND11, ZMYND11, PRNP, PRND, RASSF2, RF00026, SLC23A2, TMEM230, PCNA, CDS2, RF00377, ENSBTAG00000020668, PROKR2, GPCPD1, SHLD1, CHGB, TRMT6, MCM8, CRLS1, LRRN4, FERMT1, ENSBTAG00000046562, BMP2, ENSBTAG00000048291, ENSBTAG00000045753, ENSBTAG00000046276, ENSBTAG00000046776, ENSBTAG00000047638, ENSBTAG00000047320, ENSBTAG00000047962, ENSBTAG00000048089, ENSBTAG00000047653, ENSBTAG00000047102, ENSBTAG00000046716, ENSBTAG00000045555, ENSBTAG00000046631, ENSBTAG00000007199, HAO1, HAO1, ENSBTAG00000032156, ADRA1D, SMOX, RNF24, PANK2, bta-mir-103-2, MAVS, AP5S1, CDC25B, CENPB, SPEF1, C13H20orf27, HSPA12B, ENSBTAG00000027412, SIGLEC1, ADAM33, GFRA4, GFRA4, ATRN, C13H20orf194, C13H20orf194, C13H20orf194, SLC4A11, ITPA, DDRGK1, LZTS3, UBOX5, AVP, OXT, MRPS26, PTPRA, VPS16, PCED1A, TMEM239, C13H20orf141, CPXM1, CPXM1, EBF4, IDH3B, IDH3B, NOP56, RF00274, RF00275, RF00594, RF00432, RF00610, TMC2, SNRPB, RF00570, TGM6, TGM3, ENSBTAG00000022534, RF00156, STK35, PDYN, SIRPA, SIRPA, ENSBTAG00000048087, ENSBTAG00000039016, ENSBTAG00000039016, SIRPB1, SIRPB1, ENSBTAG00000011638, ENSBTAG00000027221, GINS1, PCMTD2, MYT1, NPBWR2, OPRL1, LKAAEAR1, RGS19, TCEA2, SOX18, PRPF6, RF00026, SAMD10, ZNF512B, ZNF512B, UCKL1, bta-mir-1388, DNAJC5, DNAJC5, TPD52L2, TPD52L2, ABHD16B, ZBTB46, SLC2A4RG, ENSBTAG00000007501, ZGPAT, ZGPAT, ARFRP1, TNFRSF6B, ENSBTAG00000047150, ENSBTAG00000047150, ENSBTAG00000047150, RTEL1, STMN3, GMEB2, FNDC11, SRMS, PTK6, PPDPF, EEF1A2, KCNQ2, KCNQ2, ENSBTAG00000031764, CHRNA4, COL20A1, COL20A1, COL20A1, ARFGAP1, ARFGAP1, BIRC7, BIRC7, YTHDF1, bta-mir-124b, RF00635, BHLHE23, SLC17A9, GID8, DIDO1, ENSBTAG00000046520, COL9A3, OGFR, MRGBP, NTSR1, SLCO4A1, bta-mir-133a-1, bta-mir-1-1, GATA5, RBBP8NL, CABLES2, RPS21, bta-mir-2306, LAMA5, ADRM1, ADRM1, OSBPL2, OSBPL2, OSBPL2, PSMA7, ENSBTAG00000021558, HRH3, MTG2, SS18L1, TAF4, TAF4, CDH4, CDH26, FAM217B, PPP1R3D, SYCP2, PHACTR3, EDN3, ZNF831, PRELID3B, ATP5F1E, TUBB1, CTSZ, NELFCD, GNAS, GNAS, GNAS, GNAS, bta-mir-296, NPEPL1, ENSBTAG00000003872, ENSBTAG00000038838, APCDD1L, VAPB, RAB22A, ANKRD60, C13H20orf85, PMEPA1, ZBP1, ZBP1, ZBP1, PCK1, CTCFL, RBM38, RAE1, SPO11, ENSBTAG00000047773, BMP7, bta-mir-2307, ENSBTAG00000037492, TFAP2C, ENSBTAG00000024608, FAM209A, RTF2, GCNT7, CASS4, RF00001, CSTF1, AURKA, FAM210B, MC3R, SIRPD, ENSBTAG00000006539, NSFL1C, FKBP1A, SDCBP2, SNPH, RAD21L1, TMEM74B, PSMF1, RSPO4, ANGPT4, FAM110A, SLC52A3, SRXN1, TCF15, CSNK2A1, TBC1D20, RBCK1, TRIB3, NRSN2, ENSBTAG00000027386, ZCCHC3, C13H20orf96, DEFB129, ENSBTAG00000048009, DEFB119, DEFB119, DEFB122A, DEFB122, DEFB123, DEFB124, REM1, HM13, ID1, COX4I2, BCL2L1, TPX2, MYLK2, FOXS1, ENSBTAG00000031214, DUSP15, TTLL9, PDRG1, XKR7, CCM2L, HCK, HCK, TM9SF4, PLAGL2, POFUT1, POFUT1, POFUT1, POFUT1, KIF3B, ASXL1, NOL4L, NOL4L, COMMD7, DNMT3B, MAPRE1, SUN5, RF00026, BPIFB2, BPIFB6, BPIFB6, BPIFB6, BPIFB3, BPIFB4, ENSBTAG00000038453, BPIFA2A, BPIFA2C, ENSBTAG00000031375, ENSBTAG00000011704, BPIFA2B, ENSBTAG00000031373, BPIFA3, BPIFA3, BPIFA1, BPIFB1, BPIFB5, ENSBTAG00000031354, CDK5RAP1, SNTA1, ENSBTAG00000014869, CBFA2T2, NECAB3, C13H20orf144, C13H20orf144, ACTL10, E2F1, PXMP4, ZNF341, ZNF341, CHMP4B, RALY, EIF2S2, RF00045, ASIP, AHCY
13:64358933-64432139<br>ITCH, ENSBTAG00000046623
13:64450162-64500844<br>ITCH, DYNLRB1, MAP1LC3A, PIGU
25:18619922-18639119<br>ACSM3
25:18672747-18714923<br>REXO5
25:18946238-18966876<br>DNAH3
**Stayability**
1:33285504-33305018<br>CADM2

3:43009131-43028743<br>CDC14A
4:55984897-56013042<br>ZNF277
11:1424088-1433201<br>ACOXL
13:1339928-13434427<br>PLCB1, PLCB4, LAMP5, PAK5, ENSBTAG00000046074, RF00408, MRPL33, ANKEF1, SNAP25, MKKS, RF00026, SLX4IP, JAG1, SRY, BTBD3, RF00156, ENSBTAG00000045631, SPTLC3, RF00273, ISM1, TASP1, TASP1, ESF1, NDUFAF5, SEL1L2, FLRT3, MACROD2, KIF16B, ENSBTAG00000032609, SNRPB2, OTOR, NANP, ENSBTAG00000023169, ENSBTAG00000004354, ENSBTAG00000022922, ENSBTAG00000022922, ENSBTAG00000040046, CCDC3, CAMK1D, RF00619, CDC123, NUDT5, SEC61A2, DHTKD1, UPF2, RF00012, PROSER2, ECHDC3, USP6NL, CELF2

Table S3. Enriched GO terms and KEGG pathways significantly enriched (≤ 0.05) from DAVID software for age at first calving.

**Age at first calving**				
**Category**	**Term**	**Genes**	***p*-Value**	**GO Term**
Biological Process	mitochondrial translational elongation	4	3,6E-2	GO:0070125
	mitochondrial translational initiation	4	3,6E-2	GO:0070124
	regulation of intracellular pH	3	3,9E-2	GO:0051453
Cell Componet	spindle pole	4	3,6E-2	GO:0000922
Molecular Function	interleukin-1, Type I, activating receptor activity	2	1,9E-2	GO:0004909
	interleukin-1 receptor activity	2	2,8E-2	GO:0004908

Table S4. Enriched GO terms and KEGG pathways significantly enriched (≤ 0.05) from DAVID software for accumulated productivity.

**Accumulated productivity**				
**Category**	**Term**	**Genes**	***p*-Value**	**GO Term**
Biological Process	positive regulation of JAK-STAT cascade	4	1,1E-4	GO:0046427
	response to organic substance	3	1,1E-3	GO:0010033
	response to hydrogen peroxide	3	7,8E-3	GO:0042542
	regulation of multicellular organism growth	3	1,2E-2	GO:0040014
	negative regulation of complement activation, classical pathway	2	1,5E-2	GO:0045959
	protein phosphorylation	5	1,9E-2	GO:0006468
	protein localization to paranode region of axon	2	2,9E-2	GO:0002175
	metabolic process	4	3,1E-2	GO:0008152
	relaxation of cardiac muscle	2	3,7E-2	GO:0055119
	relaxation of vascular smooth muscle	2	4,4E-2	GO:0060087
	paranodal junction assembly	2	4,4E-2	GO:0030913
Cell Component	other organism cell	2	1,5E-2	GO:0044216
	basolateral plasma membrane	4	3,9E-2	GO:0016323
	cytosol	14	4,3E-2	GO:0005829
Molecular function	heparin binding	5	8,2E-3	GO:0008201
	GTPase activator activity	6	1,2E-2	GO:0005096
	pancreatic polypeptide receptor activity	2	2,2E-2	GO:0001602
	[heparan sulfate-glucosamine N-sulfotransferase activity	2	3,0E-2	GO:0015016
	peptide YY receptor activity	2	3,0E-2	GO:0001601
Pathway	Complement and coagulation cascades	5	2,9E-3	-
	Cell adhesion molecules (CAMs)	6	7,7E-3	-

	MicroRNAs in cancer	6	4,8E-2	-

Table S5. Enriched GO terms and KEGG pathways significantly enriched (≤ 0.05) from DAVID software for heifer early calving until 30 months.

Heifer early calving until 30 months				
**Category**	**Term**	**Genes**	***p*-Value**	**GO Term**
Biological Process	negative regulation of cysteine-type endopeptidase activity	9	1,3E-11	GO:2000117
	innate immune response	15	9,6E-4	GO:0045087
	fatty acid alpha-oxidation	3	7,8E-3	GO:0001561
	defense response to bacterium	7	1,2E-2	GO:0042742
	negative regulation of transcription, DNA-templated	13	1,8E-2	GO:0045892
	negative regulation of sequence-specific DNA binding transcription factor activity	5	1,9E-2	GO:0043433
	neuron projection extension	3	2,6E-2	GO:1990138
	cytoskeleton-dependent intracellular transport	3	3,6E-2	GO:0030705
	microtubule-based movement	5	4,0E-2	GO:0007018
	negative regulation of fat cell differentiation	4	4,5E-2	GO:0045599
	regulation of sodium ion transmembrane transport	2	4,6E-2	GO:1902305
Cell Component	cell surface	16	7,7E-3	GO:0009986
	extracellular region	20	1,9E-2	GO:0005576
	microtubule	9	2,1E-2	GO:0005874
	extrinsic component of cytoplasmic side of plasma membrane	5	3,4E-2	GO:0031234
Molecular Function	cysteine-type endopeptidase inhibitor activity	10	7,5E-9	GO:0004869
	protease binding	10	3,0E-8	GO:0002020
	lipid binding	14	6,8E-8	GO:0008289
	oxidoreductase activity	12	5,5E-5	GO:0016491
	neurohypophyseal hormone activity	2	4,7E-2	GO:0005185
	phytanoyl-CoA dioxygenase activity	2	4,7E-2	GO:0048244
Pathway	ribosome biogenesis in eukaryotes	7	3,0E-3	-

Table S6. Enriched GO terms and KEGG pathways significantly enriched (≤ 0.05) from DAVID software for stayability.

Stayability				
**Category**	**Term**	**Genes**	***p*-Value**	**GO Term**
Biological Process	cerebral cortex development	3	2,1E-3	GO:0021987
Molecular Function	phosphatidylinositol phospholipase C activity	2	2,8E-2	GO:0004435
Pathway	Aldosterone synthesis and secretion	3	9,0E-3	-
	Insulin secretion	3	9,7E-3	-
	Sphingolipid signaling pathway	3	2,0E-2	-
	Oxytocin signaling pathway	3	3,1E-2	-

Table S7. Common gene list associated with de with reproductive traits in Nelore cattle.

Traits	Total Common Genes
AFC and STAY: 1	ACOXL
AFC and HC30: 3	RAB11FIP5, SFXN5, EMX1
HC30 and STAY: 31	SPTLC3, RF00273, ISM1, TASP1, ESF1, NDUFAF5, SEL1L2, FLRT3, MACROD2, KIF16B, ENSBTAG00000032609, SNRPB2, OTOR, NANP, ENSBTAG00000023169, ENSBTAG00000004354, ENSBTAG00000022922, ENSBTAG00000040046, CCDC3, CAMK1D, RF00619, CDC123, NUDT5, SEC61A2, DHTKD1, UPF2, RF00012, PROSER2, ECHDC3, USP6NL, CELF2
